# Evaluation of the Effects of Atorvastatin and N-Acetyl Cysteine on Platelet Counts in Patients with Primary Immune Thrombocytopenia: An Exploratory Clinical Trial

**DOI:** 10.3390/medicina59061122

**Published:** 2023-06-11

**Authors:** Lorena A Cervantes-Pérez, Gabino Cervantes-Guevara, Enrique Cervantes-Pérez, Guillermo Alonso Cervantes-Cardona, Adriana Nápoles-Echauri, Alejandro González-Ojeda, Clotilde Fuentes-Orozco, Gabino Cervantes-Pérez, Carlos A Reyes-Torres, Francisco Javier Hernández-Mora, Ana Lucia Ron-Magaña, Janet Cristina Vázquez-Beltrán, María Isabel Hernández-Rivas, Sol Ramírez-Ochoa

**Affiliations:** 1 Department of Hematology, Hospital Civil de Guadalajaras “Fray Antonio Alcalde”, Guadalajara 44280,, Mexico; lorena_tikalcerva@hotmail.com (L.A.C.-P.); lucyronhc2@gmail.com (A.L.R.-M.); 2 Department of Welfare and Sustainable Development, Centro Universitario del Norte, Universidad de Guadalajara, Colotlán 46200, Mexico; gabino_guevara@hotmail.com; 3 Department of Gastroenterology, Hospital Civil de Guadalajara “Fray Antonio Alcalde”, Guadalajara 44280, Mexico; 4 Department of Internal Medicine, Hospital Civil de Guadalajara “Fray Antonio Alcalde”, Health Sciences University Center, Universidad de Guadalajara, Guadalajara 44280, Mexico; enrique19896@msn.com (E.C.-P.); gabino-1994@hotmail.com (G.C.-P.); 5 Centro Universitario de Tlajomulco, University of Guadalajara, Tlajomulco de Zúñiga 45641, Mexico; 6 Department of Philosophical, Methodological and Instrumental Disciplines, Centro Universitario de Ciencias de la Salud, Universidad de Guadalajara, Guadalajara 44340, Mexico; gacervantes66@hotmail.com (G.A.C.-C.); izardetheodoroth@gmail.com (A.N.-E.); 7 Biomedical Research Unit 02, Specialties Hospital of the Western National Medical Center, Mexican Institute of Social Security, Guadalajara 44329, Mexico; avygail5@gmail.com (A.G.-O.); clotilde.fuentes@gmail.com (C.F.-O.); 8 School of Health Sciences, Instituto Tecnológico y de Estudios Superiores de Monterrey, Mexico City 14380, Mexico; lncarlosreyes@gmail.com; 9 Human Reproduction, Growth and Child Development Clinic, Health Sciences University Center, Universidad de Guadalajara, Guadalajara 44340, Mexico; frank.gine@gmail.com; 10 School of Medicine, Instituto Politécnico Nacional, Mexico City 11340, Mexico; janet.cris.beltran@gmail.com; 11 Departament of Odontology for the Preservation of Health, Health Sciences University Center, Universidad de Guadalajara, Guadalajara 44280, Mexico; misabel.hernandez@academicos.udg.mx

**Keywords:** immune thrombocytopenia, atorvastatin, steroids, N-acetyl cysteine

## Abstract

*Objective:* We aimed to evaluate the efficacy of the combination of atorvastatin and N-acetyl cysteine in increasing platelet counts in patients with immune thrombocytopenia who were resistant to steroid therapy or had a relapse after treatment. *Material and Methods*: The patients included in this study received oral treatment of atorvastatin at a dose of 40 mg daily and N-acetyl cysteine at a dose of 400 mg every 8 h. The desired treatment duration was 12 months, but we included patients who completed at least 1 month of treatment in the analysis. The platelet counts were measured prior to the administration of the study treatment and in the first, third, sixth, and twelfth months of treatment (if available). A *p* value < 0.05 was considered statistically significant. *Results*: We included 15 patients who met our inclusion criteria. For the total treatment duration, the global response was 60% (nine patients); eight patients (53.3%) had a complete response and one patient (6.7%) had a partial response. Six patients (40%) were considered as having undergone treatment failure. Of the responder group, five patients maintained a complete response after treatment (55.5%), three patients maintained a partial response (33.3%), and one patient (11.1%) lost their response to the treatment. All of the patients in the responder group had significant increases in their platelet counts after treatment (*p* < 0.05). *Conclusion*: This study provides evidence of a possible treatment option for patients with primary immune thrombocytopenia. However, further studies are needed.

## 1. Introduction

Immune thrombocytopenic purpura (ITP) is an acquired autoimmune disorder that features both the deficient production and destruction of platelets, resulting in a low platelet count [1]. As it is not associated with any added condition, it is classified as primary; therefore, the diagnosis is based on the exclusion of other causes of thrombocytopenia [1]. This condition has variable clinical symptoms, such as bleeding, bruising, and petechiae, and it affects the quality of life of patients related to the restriction of activities, anxiety about the risk of bleeding, fatigue, and the burden of treatment and follow-ups [1].

Steroids are the gold standard for the management of patients with primary immune thrombocytopenia [2,3]; nevertheless, one-third of patients relapse or do not achieve a complete response [3,4,5]. These patients can be classified as resistant to corticosteroid therapy if they do not show a response after at least 3 months of treatment, and a second-line therapy should be considered according to the patient’s clinical situation [1].

The extended use of steroids leads to major adverse events; for this reason, the American Society of Hematology recommends a variety of second-line treatments, including thrombopoietin receptor agonists (TPO-RA) (eltrombopag, romiplostim, and avatrombopag), rituximab, and splenectomy, with response rates per month of 65.7%, 86.7%, and 62.1%, respectively [1]. The decision to choose which therapy to follow must be individualized and will depend on the duration of ITP, the frequency of bleeding episodes that require hospitalization or rescue medication, comorbidities, the age of the patient, adherence to the medication, medical and social support networks, patient values and preferences, cost, and availability; the complications of each therapy should also be considered, that is, whether the long-term benefits are greater than the risks [1]. Both TPO-Ras and rituximab are high-cost treatments with limited access, especially in developing countries such as Mexico [2,3,4,6]; on the other hand, a splenectomy is an irreversible procedure that carries operation risks as well as risks of infection and thrombosis [1].

The drugs used in this study were atorvastatin and N-acetyl cysteine, both of which are worth discussing for their off-label uses.

Atorvastatin is used for oral dyslipidemia and reversibly and competitively inhibits the enzyme hydroxymethylglutaryl-coenzyme A (HMG-CoA) reductase, whose metabolic reaction catalyzes its conversion to mevalonic acid, ultimately affecting cholesterol, low-density lipoproteins (LDLs), and very low-density lipoproteins (VLDLs), thus reducing the cardiovascular risk [7,8]. Although the main indication for this drug is lipid reduction, it has been seen that it may play a therapeutic role both in erectile dysfunction and cancer management with antitumor pathways that inhibit its proliferation and survival; it has the ability to treat chronic diseases of the respiratory tract [9,10,11].

On the other hand, N-acetyl cysteine is a drug derived synthetically from the endogenous amino acid L-cysteine that is used for paracetamol overdoses by inactivating the toxic metabolite of paracetamol, N-acetyl-p-benzoquinoneimine (NAPQI), and acting as a mucolytic agent; in addition to its antioxidant properties (as an oxidative stress mitigator), it also treats severe alcoholic hepatitis; prevents contrast-induced nephropathy; treats psychiatric disorders, doxorubicin-induced cardiotoxicity, AIDS, and neurological affectations; increases the production of nitric oxide, which causes vasodilation, increasing the supply and absorption of oxygen to and by tissues; and, finally, has an anti-inflammatory effect [12,13,14,15].

However, even though these drugs are widely used and have a good safety profile, several adverse events have been reported; for example, atorvastatin can raise liver transaminases more than three times the upper limit of what is normal as well as the rare elevation in creatinine and phosphokinase as a reflection of rhabdomyolysis, and it can also cause a decrease in one’s platelet levels [8,16].

N-acetyl cysteine can cause gastrointestinal symptoms (nausea, vomiting, diarrhea, flatulence, and reflux), chest pain, hypotension, respiratory distress, headache, lethargy, and fever and even abnormalities in an electrocardiogram, an epileptic status, a disease similar to serum sickness, and anaphylactoid reactions in 18% of patients both after oral and intravenous administration [17,18,19,20].

Nevertheless, atorvastatin and N-acetyl cysteine are two widely available drugs with good and safe pharmacological profiles [21,22,23] that are easily available for our population of patients. The evidence supports the effects of atorvastatin on endothelial progenitors, which include the improvement of their mobilization and the improvement of peripheral blood circulation [7,8]. Recently, N-acetyl cysteine, in addition to its membrane stabilizer and antioxidant abilities [6], has been demonstrated to stimulate the function and number of mesenchymal cells in the bone marrow, and it secondarily influences megakaryopoiesis [24,25].

The objective of this study was to evaluate the efficacy of the combination of atorvastatin and N-acetyl cysteine in increasing platelet counts in patients with immune thrombocytopenia who were resistant to steroid therapy or had a relapse after treatment.

## 2. Materials and Methods

For this study, we included patients with primary immune thrombocytopenia who were refractory to treatment or in relapse, who were admitted to our hospital between July 2018 and December 2019, and who met the following inclusion criteria: (a) men or women aged older than 15 years, (b) a diagnosis of thrombocytopenia (platelet count < 100 × 10^9^/L), (c) a diagnosis of primary immune thrombocytopenia that relapsed or was refractory to primary treatment with steroids, and (d) taking steroids to treat immune thrombocytopenia at a stable dose at the time of inclusion (dose unchanged in the last 4 weeks). The included patients also did not meet any of the exclusion criteria: (a) a diagnosis of secondary immune thrombocytopenia; (b) pregnancy; (c) hypersensitivity to any of the study drugs; (d) undergoing anticoagulant treatment or taking medications that could alter one’s platelet count; (e) liver enzyme abnormality defined as an elevation in AST or ALT above three times the upper limit; (f) elevation in creatinine above 1.5 mg/dl or known diagnosis of chronic kidney disease; and (g) a diagnosis of chronic diseases that, at the discretion of the investigator, disqualify them from participating in this study.

Patients who continuously received steroid treatment for at least 10 weeks without significant elevation in serum platelets were considered resistant to steroid treatment. Patients considered to have relapsed presented resistance to steroid treatment despite having a history of response in one or more prior episodes.

All the patients included in this study continued with their base treatment (corticosteroids) accompanied by a second-line treatment (azathioprine or azathioprine/danazol).

Clinical, physical, and biochemical evaluations were performed on all patients included in this study. Platelet counts were measured prior to study treatment and in the first, third, sixth, and twelfth months of treatment (if available).

The patients included in this study received oral treatment of atorvastatin at a dose of 40 mg daily and N-acetyl cysteine at a dose of 400 mg every 8 h. The desired treatment duration was 12 months, but we included patients who completed at least 1 month of treatment in the analysis.

The response to treatment was classified as follows: (a) a partial response, where the patient reached a platelet count above 30 × 10^9^/L or had an increase to at least double the platelet count compared to the baseline count, or (b) a complete response, where patients reached a platelet count above 100 × 10^9^/L. Global response refers to the percentage of the total number of patients who achieved a partial or global response.

For data processing and the descriptive and inferential statistical analyses, we used the software IBM SPSS statistics (IBM, Armonk, NY, USA).

We calculated the absolute and relative frequencies, which are presented as percentages and proportions, for the descriptive analysis of the qualitative variables. The quantitative variables were analyzed using their means, standard deviations, medians, and ranges. For the inferential analysis of the quantitative variables, the chi-square test was performed to make the comparison using a contingency table; for the inferential analysis of the quantitative variables, their normality in the Gaussian distribution was first assessed using the Shapiro–Wilk test, and then a comparison of nonpaired groups was performed with the Student’s *t*-test for parametric data and with the Mann–Whitney U test for nonparametric data. A *p*-value < 0.05 was considered statistically significant.

This study was conducted in accordance with the Declaration of Helsinki. This study was approved by the Ethics Committee of the Hospital Civil de Guadalajara Fray Antonio Alcalde (R-179/19), and informed consent was obtained from each patient before any procedures were performed in this study. This trial is registered at clnicaltrails.gov under trial number NCT05551624.

## 3. Results

We included 15 patients who met our inclusion criteria. The demographic characteristics of the cohort are shown in Table 1.

In the first month of treatment, the global response was 26.6% (*n* = 4), with all complete responses, and 73.3% of the patients (*n* = 11) were considered to be non-responders to the treatment in this time frame.

In the third month of treatment, only 13 patients continued the treatment. The global response was 30.8% (*n* = 4); three patients (23%) had a complete response, and one patient (7.8%) had a partial response. Nine patients (69.2%) were considered to have no response to the treatment.

At six months of treatment, only seven patients continued the treatment. The global response was 57.1% (*n* = 4); three patients (42.8%) had a complete response, and one patient (14.4%) had a partial response. Three patients (42.9%) were classified as non-responders to the treatment.

In the twelfth month of treatment, only four patients continued the treatment. The global response was 100%; three patients (75%) had a complete response, and one patient (15%) had a partial response.

For the total treatment duration, the global response was 60% (nine patients); eight patients (53.3%) had a complete response, and one patient (6.7%) had a partial response. Six patients (40%) were considered to have undergone treatment failure.

Adherence to the treatment was maintained during the follow-up. No adverse events related to the study treatment were noted throughout the duration of this study.

For further analysis, we divided the cohort into two groups: (1) responders to treatment and (2) non-responders to treatment. In Table 2, we compare the platelet counts of the responder and non-responder groups before and after treatment.

In the responder group, four patients maintained a complete response after treatment (55.5%), three patients maintained a partial response (33.3%), and one patient (11.1%) lost their response to the treatment.

In Table 3, we compare the clinical and demographic characteristics of the two groups (responders vs. non-responders).

## 4. Discussion

Although the first line of treatment (steroid-based) for primary immune thrombocytopenia is effective in most patients [2,3,4,26], a significant number of patients are resistant to treatment or present with frequent relapses [4,5,27].

The efficacy of atorvastatin plus N-acetyl cysteine was substantiated because a dysfunction in the endothelial progenitors was demonstrated in patients with immune thrombocytopenia who were resistant to steroids [22] as well as an increase in oxidative stress [28,29].

In a study by Kong and col. [22], results similar to ours were obtained for patients with primary immune thrombocytopenia who were resistant to treatment with steroids, and the global response to treatment with atorvastatin plus N-acetyl cysteine was 69% as compared with our result of 60%. Added to the epidemiological similarity to our group of patients, in their non-responder group, a decrease in platelet counts [22] was noted, which we also found in our study (Table 2). Reports on thrombocytopenia associated with atorvastatin treatment are limited [30] but cannot be discarded. This could be due to the phenomenon of idiosyncratic drug reactions [31,32]. These adverse reactions can be classified as intrinsic (type A) or idiosyncratic (type B) [31,32]. The former are the predictable, dose-related, and toxic effects of the drug, while the latter are associated with the drug, patient, and environmental risk factors, are dose-independent, and can be difficult to predict since they do not occur within the range of doses used clinically in all people [31,32]; they are also subdivided into immunological (hypersensitivity, allergic, and immune reactions) or nonimmunological (caused by alterations in the metabolic pathways that form a toxic compound) [31,32]. A few cases of idiosyncratic reactions related to atorvastatin have been reported. Narayanan et al. [30] describe a male patient with ischemic heart disease who received atorvastatin for the management of hypercholesterolemia and presented an episode of thrombocytopenic purpura which resolved when this drug was discontinued, showing a gradual improvement in his serum platelet count. Ghuman et al. [33] reported a female patient who developed refractory thrombocytopenia after the use of atorvastatin for the treatment of hyperlipidemia, presenting to medical services with atraumatic hematomas and a low platelet count, and who finally achieved an increase in these upon the discontinuation of atorvastatin and the management of thrombocytopenia.

All the patients in the responder group in this study showed significant increases in their platelet counts compared to the counts prior to the study treatment, and even with our limited sample, we can begin to see a tendency for a higher proportion of better responses related to the duration of treatment exposure.

It is worth mentioning that the patients who did not continue the study all corresponded to the non-responders group, so the overall response results should be interpreted with caution as well as the differences reported in the mean platelet count after treatment since the non-responder group was not exposed to the study drug for the same proportion of time. The reason for the withdrawal of these patients from this study was due to the withdrawal of consent and patient safety.

To our knowledge, this is the first study using this treatment for primary immune thrombocytopenia in our country, and only a few studies have been reported to date in other parts of the world (most of which were performed in China) [22,34,35].

Even though second-line treatment options are well established for the treatment of this patient population [1], access to these drugs is quite limited in developing countries; for example, in our population, public health institutions do not provide patients with established second-line drugs (rituximab or TGO-RA), so patients must access them independently and at high costs. This is one of the reasons why drugs such as azathioprine and danazol, even with their limited clinical evidence of effectiveness [6,36], continue to be used in this sociodemographic context.

## 5. Conclusions

Although this is an exploratory study, we obtained promising results showing a good response to a treatment that is easily accessible even in countries under development, and the results showed that this treatment could be a good option to include in the established treatments for this disease.

Some of the limitations of our study were the small sample size of the patients, the absence of a control group for the treatment, and the low and imbalanced retention of patients for follow-up and analysis. In further studies, we need to evaluate the clinical efficacy of the treatment, including more than the platelet counts, and a study needs to be designed with a control group, with randomization, and with a larger homogeneous sample. In conclusion, even with the aforementioned limitations, the overall response to the test treatment in this study was around 60%, and a trend towards a better response can be observed in relation to the exposure time of the study drug, opening the possibility of a future therapeutic option that would be worth studying in greater depth in the future and that would provide a cost-effective option, which is especially necessary in developing countries.

## Figures and Tables

**Table 1:** Demographic characteristics of the patients.

Total Patients (*n*)	15
Sex	
Female, *n* (%)	13 (86.6)
Male, *n* (%)	2 (13.3)
Mean age at inclusion, years (range)	48.06 (16.67)
Follow-up duration, days (range)	157.53 (90.60)
Global mean platelet count, ×10^9^/L (range)	
Initial, *n* = 15	45.91 (17.58)
1 month, *n* = 15	82 (67.04)
3 months, *n* = 13	66.74 (39.64)
6 months, *n* = 7	104.88 (63.75)
1 year, *n* = 4	108.38 (27.65)
Previous treatment line, *n* (%)	
Methylprednisolone	6 (40)
Prednisone	13 (86.6)
Dexamethasone	10 (66.6)
Azathioprine	15 (100)
Danazol	3 (20)
Romiplostim	3 (20)
Medications during the protocol, *n* (%)	
Prednisone	10 (66.6)
Dexamethasone	1 (6.6)
Azathioprine	15 (100)
Danazol	2 (13.3)
Comorbidities, *n* (%)	
Diabetes mellitus	4 (26)
Systemic arterial hypertension	6 (40)
Dyslipidemia	1 (6)
Iron-deficiency anemia	3 (20)
Uterine myomatosis	2 (13)

*n* = number of patients.

**Table 2:** Platelet counts of the responder group and non-responder group before and after treatment.

Responder Group			
Treatment Evaluation	Mean Platelet Counts before the Intervention, ×10^9^/L	Mean Platelet Counts after the Intervention, ×10^9^/L	*p*
GLOBAL (range), *n* = 6	46 (19–65)	149 (42–251)	0.0009 *
1 month (range), *n* = 4	46 (34–65.5)	168 (65–251)	0.0295 *
3 months (range), *n* = 4	41 (19–65)	98 (42–142)	0.0424 *
6 months (range), *n* = 4	47 (35–65)	143 (57–221)	0.0295 *
12 months (range), *n* = 4	58 (45–65)	106 (70–133)	0.0276 *
Non-responder Group			
Treatment evaluation	Mean platelet counts before the intervention, ×10^9^/L	Mean platelet counts after the intervention, ×10^9^/L	*p*
GLOBAL, *n* = 6	46 (7–62)	45 (8–57)	0.9472
1 month (range), *n* = 4	45 (19–65)	46 (12–72)	0.6072
3 months (range), *n* = 4	49 (7–65)	53 (5–86)	0.5915
6 months (range), *n* = 4	60 (45–72)	52 (38–62)	0.1519

*n* = number of patients; * *p* < 0.05.

**Table 3:** Baseline characteristics of the responder and non-responder groups and comparison of the platelet counts of both groups.

	With a Response	With No Response	*p*
Number of patients	9	6	
Sex, *n*			
Female	7	6	0.2148
Age, years, mean (SD)	46.11 (15.02)	51 (20.02)	0.597
Previous lines of treatment, median (range)	3.44 (2–5)	3.66 (2–6)	0.7982
Follow-up time, mean days (SD)	177.4 (104)	127.7 (62.37)	0.3146
Previous relapses, median (range)	0 (0–3)	2.5 (0–3)	0.0573
Mean response time, median days (range)	41 (20–310)	13.5 (0–92)	0.1514
Response duration, mean months (range)	3.2 (1.8–6)	N/A	
Adverse effects, total	0	0	
Mean platelet counts (×10^9^/L) after treatment, *n* = 15 (range)	149 (42–251)	45 (5–85)	0.002 *
Platelet count (×10^9^/L) subanalysis by treatment time			
Baseline, median (range), *n* = 15	45 (19–475)	51.25 (7–62.50)	0.8641
1 month, mean (SD), *n* = 15	103.9 (73.11)	35.78 (32.50)	0.0533
3 months, mean (SD), *n* = 13	69.31 (46.03)	49.28 (36.52)	0.4292
6 months, mean (SD), *n* = 7	115.9 (62.09)	38.70 (N/A ♦)	0.3017
12 months, mean (SD), *n* = 4	108.37 (27.65)	N/A ϕ	

*n* = number of patients, * *p* < 0.05, SD = standard deviation, ♦ = there was only one non-responder at this stage of the study, and ϕ = no data for participants in the non-responder group.

## Data Availability

The data presented in this study are available from the corresponding author upon request. The data are not publicly available due to patient privacy.
